# H_2_O_2_ concentration-dependent kinetics of gene expression: linking the intensity of oxidative stress and mycobacterial physiological adaptation

**DOI:** 10.1080/22221751.2022.2034484

**Published:** 2022-02-16

**Authors:** Mengying Wu, Wenyan Shan, Guo-Ping Zhao, Liang-Dong Lyu

**Affiliations:** a Key Laboratory of Medical Molecular Virology of the Ministry of Education/Ministry of Health (MOE/NHC), School of Basic Medical Sciences, Fudan University, Shanghai, People’s Republic of China; b Department of Microbiology, School of Life Sciences, Fudan University, Shanghai, People’s Republic of China; c Shanghai Clinical Research Center for Infectious Disease (Tuberculosis), Shanghai Key Laboratory of Tuberculosis, Shanghai Pulmonary Hospital, Shanghai, People’s Republic of China

**Keywords:** Oxidative stress, *Mycolicibacterium smegmatis*, hydrogen peroxide, expression dynamics, antioxidant defence

## Abstract

Defence against oxidative stress is crucial for *Mycobacterium tuberculosis* to survive and replicate within macrophages. Mycobacteria have evolved multilayer antioxidant systems, including scavenging enzymes, iron homeostasis, repair pathways, and metabolic adaptation, for coping with oxidative stress. How these systems are coordinated to enable the physiological adaptation to different intensities of oxidative stress, however, remains unclear. To address this, we investigated the expression kinetics of the well-characterized antioxidant genes at bacteriostatic H_2_O_2_ concentrations ranging from 1 mM to 10 mM employing *Mycolicibacterium smegmatis* as a model. Our results showed that most of the selected genes were expressed in a H_2_O_2_ concentration-dependent manner, whereas a subset exhibited sustained induction or repression without dose–effect, reflecting H_2_O_2_ concentration-dependent physiological adaptations. Through analyzing the dynamics of the coordinated gene expression, we demonstrated that the expressions of the H_2_O_2_ scavenging enzymes, DNA damage response, and Fe–S cluster repair function were strikingly correlated to the intensity of oxidative stress. The sustained induction of *mbtB*, *irtA*, and *dnaE2* indicated that mycobacteria might deploy increased iron acquisition and error-prone lesion bypass function as fundamental strategies to counteract oxidative damages, which are distinct from the defence tactics of *Escherichia coli* characterized by shrinking the iron pool and delaying the DNA repair. Moreover, the distinct gene expression kinetics among the tricarboxylic acid cycle, glyoxylate shunt, and methylcitrate cycle suggested that mycobacteria could dynamically redirect its metabolic fluxes according to the intensity of oxidative stress. This work defines the H_2_O_2_ concentration-dependent gene expression kinetics and provides unique insights into mycobacterial antioxidant defence strategies.

## Introduction

Despite the availability of vaccine and chemotherapy, *Mycobacterium tuberculosis* (*Mtb*) remains the most successful bacterial pathogen. Among the factors that contribute to the success of *Mtb*, the ability to counteract reactive oxygen species (ROS) generated by macrophages is crucial for its survival and replication within the host [1]. In addition, increasing evidence shows that ROS-mediated oxidative stress and DNA damage contribute substantially to cell death caused by most bactericidal antibiotics in mycobacteria, indicating that antioxidant response also affects the antibiotic efficacy [2–7]. Therefore, systematic understanding of the mechanisms deployed by mycobacteria to defence against oxidative stress will promote the development of novel anti-tuberculosis strategies.

The toxicity of ROS mainly stems from the reaction of superoxide (O_2_^–^) or H_2_O_2_ with iron from mononuclear iron enzymes and the [4Fe–4S] clusters of dehydratases, resulting in inactivation of enzymes involved in various metabolic pathways, including the syntheses of branched-chain and aromatic amino acids, respiration, ribonucleotide reduction, and the tricarboxylic acid (TCA) cycle [8–10]. Auxotrophy for branch-chain amino acids and inability to grow on TCA cycle substrates were observed in *Escherichia coli* mutant strains deficient in superoxide dismutase (SOD) or catalase/peroxidase [11]. Hydrogen peroxide could also react with the pool of loose iron via the Fenton reaction and form extremely reactive and damaging hydroxyl radicals (OH^·^), which contributes substantially to ROS-induced DNA damage [12]. Hydroxyl radicals can directly react with the sugar–phosphate backbone and the base moiety within the double helix, leading to double-strand breaks (DSBs) during the process of DNA repair [13]. In addition, due to chelation of Fe^2+^ by triphosphates, H_2_O_2_ could also react with the pool of nucleotides, resulting in DNA damage upon being incorporated into DNA by polymerase [3,11]. Moreover, accumulation of damaged proteins resulted from the oxidation of cysteine and methionine residues was also observed in *E. coli* exposed to H_2_O_2_ [14]. Given the common chemical nature of ROS toxicity on cellular components, it is not surprising that bacteria have evolved a highly conserved multilayer antioxidant systems, including scavenging enzymes, maintenance of iron homeostasis, DNA and protein repair and metabolic adaptation, for coping with oxidative stress [11,15–17].

Despite the well-established role in mycobacterial antioxidant response and pathogenesis, how these antioxidant systems are coordinated to enable the physiological adaptation to different intensities of oxidative stress remains unclear [17,18]. For instance, due to the pivotal role of iron metabolism in the action of ROS, the activity of iron acquisition could not only determine the intracellular iron concentration and the repair of iron-cofactored enzymes, but also positively correlate with the severity of DNA damage [15]. In *E.coli*, it was proposed that the antioxidant response would shrink the loose-iron pool and thereby alleviate the Fenton reaction and DNA damage [15,16]. It remains unclear that how mycobacteria maintain the balance between iron metabolism and DNA damage under oxidative stress. In addition, although the toxicity of ROS is a dose-dependent event, the physiological states and the adaptation strategies under different intensities of oxidative stress remain elusive. This could be exemplified by a recent study indicating that the regulation of Clp protease activity under oxidative stress was associated with the intensity of oxidative stress [19].

In this study, using *Mycolicibacterium smegmatis* (*Msm*) as a model strain, we defined the expression kinetics of the well-characterized antioxidant genes at bacteriostatic H_2_O_2_ concentrations ranging from 1 mM to 10 mM. Our real-time RT-PCR (qRT-PCR) results revealed both H_2_O_2_ concentration-dependent expression and steadily sustained induction or repression of antioxidant genes regardless of H_2_O_2_ concentration, demonstrating H_2_O_2_ concentration-dependent physiological transitions and adaptations. Through analyzing the dynamics of coordinated gene expression, we provided several unique insights into mycobacterial antioxidant defence strategies under different intensities of oxidative stress.

## Results and discussion

**Figure 1.**
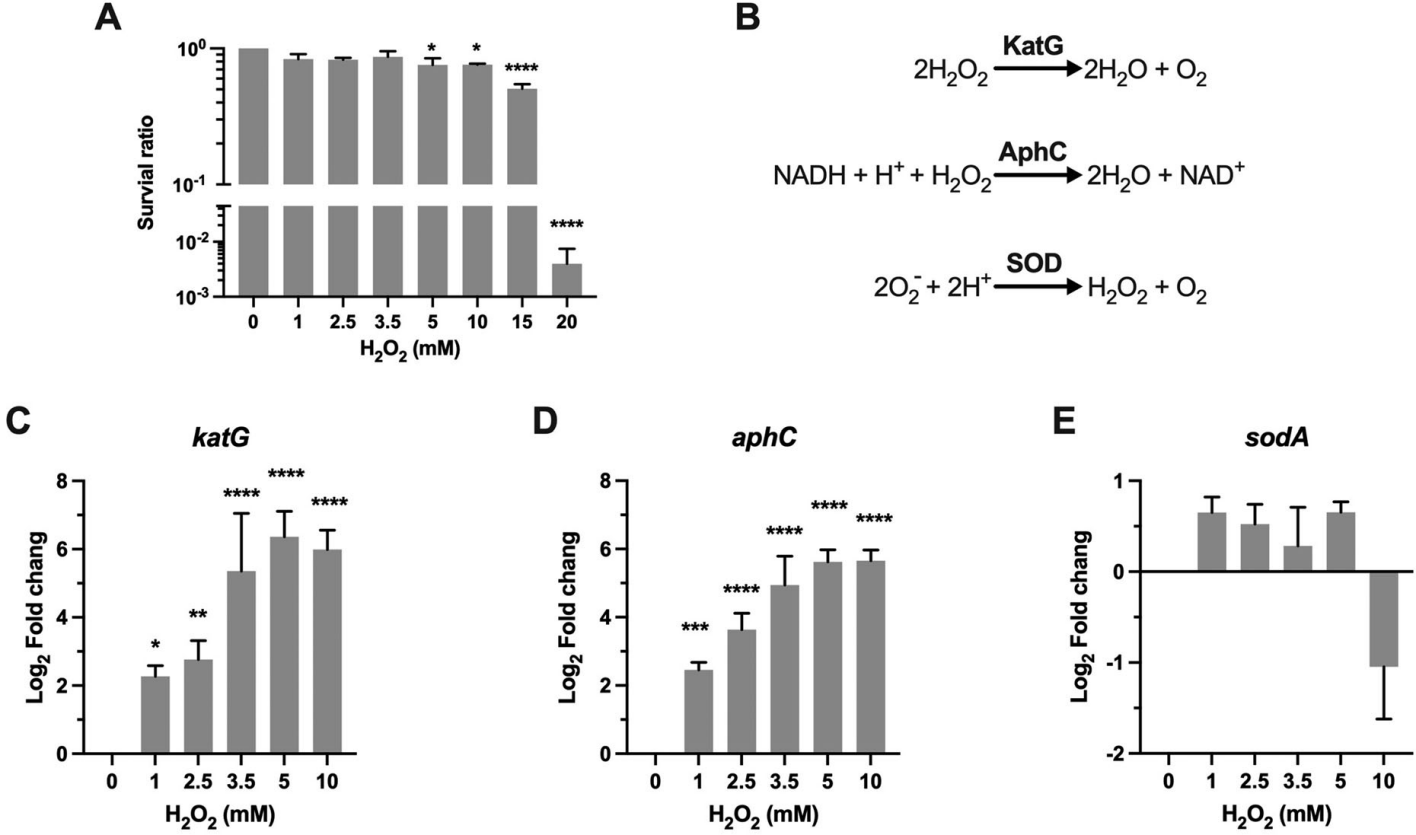
Transcriptional profiles of genes encoding for scavenging enzymes. (A) Survival of *Msm* treated with H_2_O_2_. Exponential-phase *Msm* cultures were treated with different H_2_O_2_ concentrations for 50 min, survival was measured by tenfold serial dilution plating. (B) Scavenging enzymes that are responsible for degrading ROS. (C–E) Expression of *katG*, *aphC*, and *sodA* in *Msm* exposed to H_2_O_2_ for 50 min. Transcript levels were measured by the qRT-PCR, normalized relative to *sigA*, and expressed as Log_2_ fold change from untreated cultures. Data shown are mean ± SE with at least three independent experiments. *P < 0.05, **P < 0.01, ***P < 0.001, ****P < 0.0001.

H_2_O_2_ can induce bacteriostatic or bactericidal effect, depending on the exposed H_2_O_2_ concentrations. We measured the survival of *Msm* after being exposed to H_2_O_2_ concentrations ranging from 1 to 20 mM for 50 min. The H_2_O_2_ concentrations used in this study were validated by the peroxide assay. Our results showed that H_2_O_2_ at 1–10 mM caused only a slight survival defect (killing of 13–25% *Msm* cells), while exposure to 15 and 20 mM H_2_O_2_ resulted in killing of 50% and 99.6% *Msm* cells (*P *< 0.0001), respectively (Figure 1A). The observed bacteriostatic H_2_O_2_ concentration on *Msm* (≤10 mM) is consistent with the observations on *Mtb* [20], suggesting that *Msm* and *Mtb* may deploy similar mechanisms to adapt to oxidative stress. To minimize the effect of cell death on the gene expression dynamics, H_2_O_2_ concentrations ranging from 1 to 10 mM were selected in the qRT-PCR experiments. To interrogate mycobacterial physiological states and adaptation strategies under different intensities of oxidative stress, we measured the expression dynamics of the well-characterized antioxidant genes that carry out the committed antioxidant reactions and were functionally validated in mycobacteria and/or *E.coli* (Table S1).

### Scavenging enzymes

A hallmark of the aerobic bacteria response to oxidative stress is the induction of scavenging enzymes, which can timely reduce the concentration of ROS and thus prevent the oxidative damages to cellular components (Figure 1B) [11]. Mycobacterial ROS scavenging enzymes include catalase KatG [21], the alkyl hydroperoxidase AhpC [22], and superoxide dismutases SodA and SodC [23]. All of these genes contribute to mycobacterial resistance against ROS and are well-established virulence factors [21,23–28]. KatG and AhpC can directly target and detoxify H_2_O_2_ (Figure 1B). Our qRT-PCR results demonstrated that the induction of *katG* and *ahpC* was steadily elevated from ∼4-fold (*P *< 0.05) at 1 mM H_2_O_2_ to ∼64-fold (*P *< 0.0001) at 5 mM H_2_O_2_, and no further induction was observed at 10 mM H_2_O_2_ (Figure 1C-D). This H_2_O_2_ concentration-dependent kinetics of *katG* and *ahpC* expression indicates that the committed transcriptional regulation could response sophistically via tittering the concentration of intracellular H_2_O_2_.

SodA belongs to the Fe-SOD family and accounts for a major portion of the superoxide dismutase activity of *Mtb* (Figure 1B) [29]. Although SOD do not directly react with H_2_O_2_, *Mtb* mutants with reduced *sodA* expression became extremely sensitive to H_2_O_2_ and were severely attenuated in mice [24]. We therefore analyzed the expression of *Msm sodA* and the results showed that its expression was slightly increased at the H_2_O_2_ concentrations ranging from 1 to 5 mM. Moreover, in contrast to the sustained induction of *katG* and *ahpC* at 10 mM H_2_O_2_, expression of *sodA* was downregulated ∼2-fold at this H_2_O_2_ concentration (Figure 1E). Overall, the expression pattern of *Msm sodA* is consistent with the previous studies showing that the expression of *sodA* and *sodC* was not remarkably changed in *Mtb* exposed to H_2_O_2_ [20,30].

### Iron utilization

**Figure 2.**
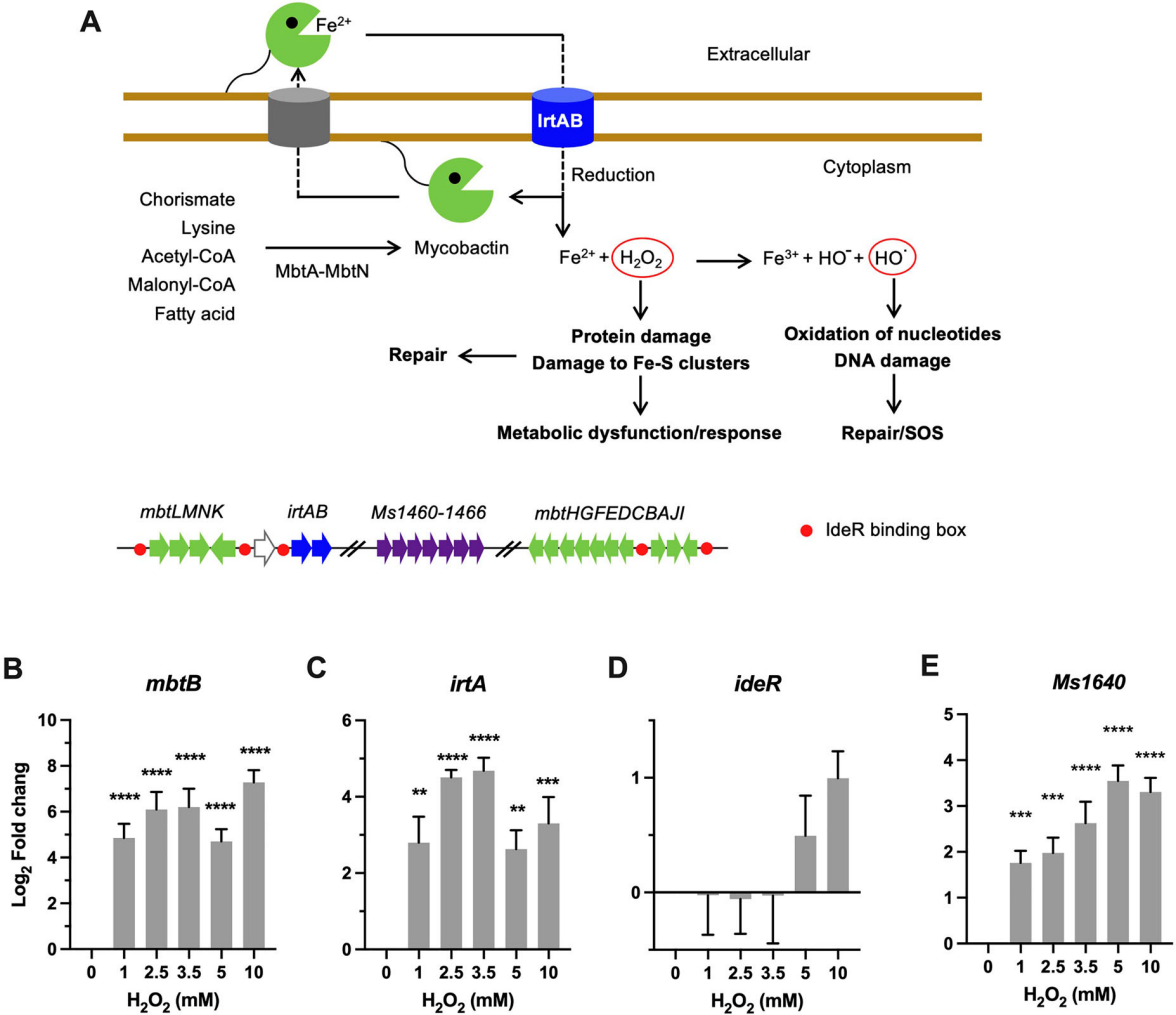
Transcriptional profiles of genes encoding for iron utilization. (A) Carton illustration of mycobacterial Fe acquisition system and its role in oxidative stress. Ms 1460–1466 are the *M. smegmatis* homologues of *Mtb* Rv1460-1466 cluster. (B–E) Expression of *mbtB*, *irtA*, *ideR*, and *Ms1640* in *Msm* exposed to H_2_O_2_ for 50 min. Transcript levels were measured by the qRT-PCR, normalized relative to *sigA*, and expressed as Log_2_ fold change from untreated cultures. Data shown are mean ± SE with at least three independent experiments. **P < 0.01, ***P < 0.001, ****P < 0.0001.

The toxicity of ROS mainly stems from the reaction of superoxide (O_2_^–^) and H_2_O_2_ with iron [8–10]. Therefore, maintaining iron homeostasis plays a pivotal role in bacterial defences against oxidative stress (Figure 2A)[16]. We examined transcriptional profiles of well-characterized genes representative of iron scavenge (*mbtB*), import (*irtA*), regulation (*ideR*), and Fe–S cluster repair (*suf*) [31–35].

Mycobacteria rely on siderophore molecules called mycobactins for the acquisition of extracellular iron [34]. Biosynthesis of mycobactin is carried out via a system encoded by 14 genes located in two *mbt* gene clusters (*mbtA-J* and *mbtK-N*) (Figure 2A). The qRT-PCR results demonstrated that the expression of *Msm mtbB* was markedly induced by 52-fold (*P *< 0.0001) by 1 mM H_2_O_2_ and remained elevated at the tested H_2_O_2_ concentrations (Figure 2B). In line with this, the expression of *irtA*, which encodes a virulence factor involved in import of Fe-carboxymycobactin [31,36], was also significantly induced 8- to 27-fold (*P *< 0.01) by the tested H_2_O_2_ concentrations (Figure 2C). Thereafter, these expression profiles appear to argue that mycobacteria tend to upregulate the iron acquisition system upon oxidative stress [20,30]. This is strikingly different from the defence strategy of *E. coli* against oxidative stress, which was characterized by shrinking the iron pool, shown by the increased sequestration of unincorporated iron and repression of iron uptake [16]. Intriguingly, the expression of *ideR*, a functional counterpart of the *E. coli* Fur repressor of iron-dependent genes including *irtA* and *mbt* clusters (Figure 2A) [33,35], was induced by 10 mM H_2_O_2_ (∼2-fold) (Figure 2D). However, it seems the induction of *ideR* did not result in repression of *irtA* and *mbt* (Figure 2B-D).

In *E.coli*, the Suf (mobilization of sulphur) system is induced by OxyR under oxidative stress to assemble and repair Fe–S clusters [11]. In mycobacteria, this system is encoded by the *suf* operon (*Ms1640*-*1466*, represent the counterparts of *Mtb* Rv1460-1466 cluster) and the *Msm* mutants with inactivated *suf* displayed growth deficiency under low-iron conditions (Figure 2A) [32]. Our qRT-PCR results demonstrated that the expression of *Ms1640* steadily increased from 4-fold at 1 mM H_2_O_2_ to 13-fold at 5 mM H_2_O_2_ (*P *< 0.0001), and no further induction was observed at 10 mM H_2_O_2_ (Figure 2E).

### DNA repair

**Figure 3.**
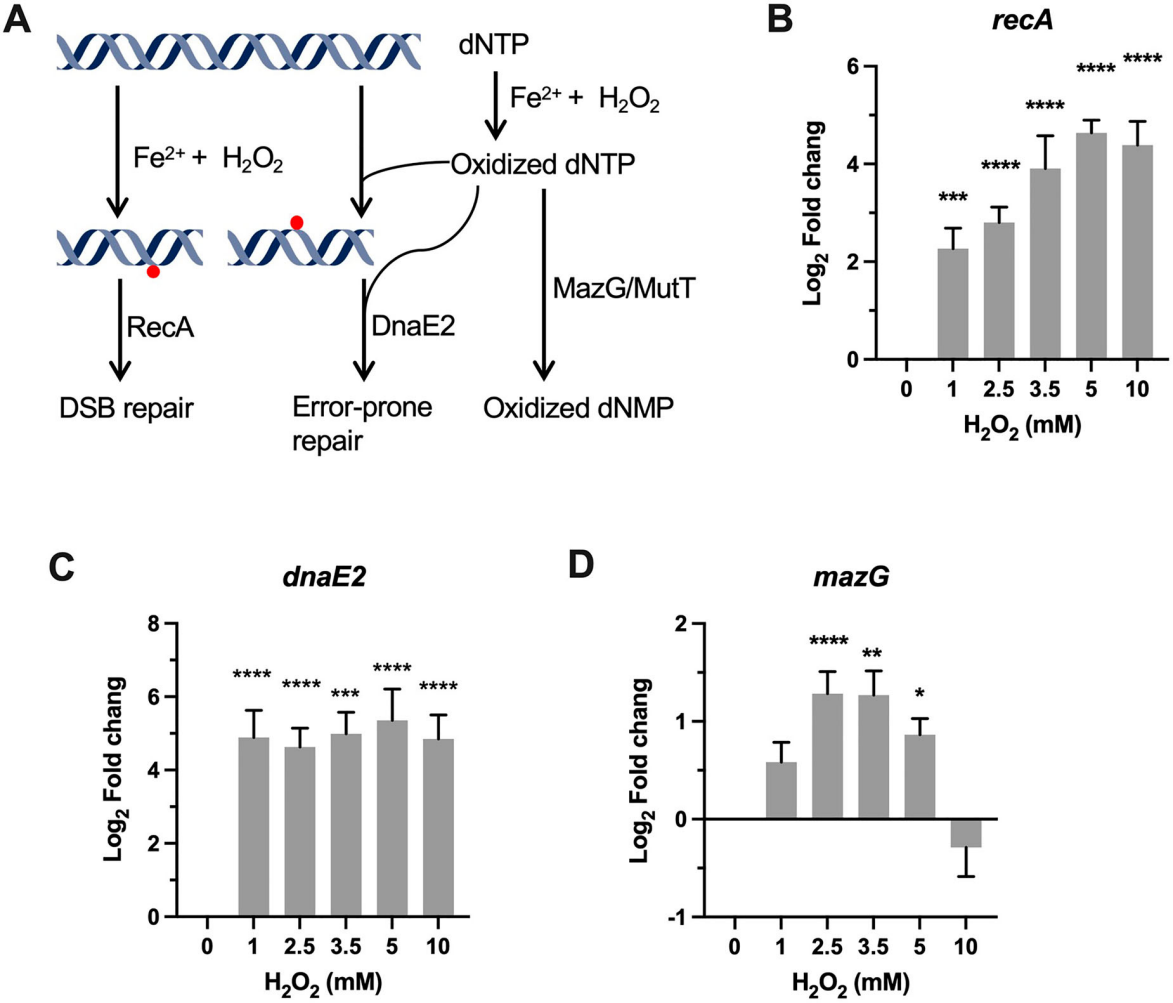
Transcriptional profiles of genes encoding for DNA repair proteins. (A) Illustration of H_2_O_2_-mediated damage to DNA and the repair systems. (B–D) Expression of *recA*, *dnaE2*, and *mazG* in *Msm* exposed to H_2_O_2_ for 50 min. Transcript levels were measured by the qRT-PCR, normalized relative to *sigA*, and expressed as Log_2_ fold change from untreated cultures. Data shown are mean ± SE with at least three independent experiments. *P < 0.05, **P < 0.01, ***P < 0.001, ****P < 0.0001.

DNA damage is the major event underlying ROS-induced cell death [12]. H_2_O_2_-induced DNA damage is mainly mediated by hydroxyl radicals (OH^·^) generated via the Fenton reaction, which are extremely reactive with both base and ribose moieties of the DNA (Figure 3A). One of the main difference in the H_2_O_2_ susceptibility between mycobacteria and *E.coli* appears to be the highly refractory of mycobacteria to DNA damage-dependent, mode-one killing caused by low concentrations of H_2_O_2_ (1–2 mM) [20,37]. The mechanism underlying this remains elusive. It was suggested that the thick cell wall contributes to the intrinsic resistance of *Mtb* to ROS [17]. However, our qRT-PCR showed that the expression of *recA*, a key DNA damage-induced factor involved in recombination, DNA repair, and induction of SOS response, was significantly induced 5.5-fold (*P *< 0.001) by 1 mM H_2_O_2_ (Figure 3B), similar to that as observed in *E.coli* [38]. Moreover, the induction of *recA* was apparently H_2_O_2_ dose-dependent, approaching 27-fold (*P *< 0.0001) increase at 5 and 10 mM H_2_O_2_ (Figure 3B), suggesting that DNA damage is correlated with the intensity of oxidative stress. These results suggest that, despite the lack of mode-one killing, DNA damage could be induced by low H_2_O_2_ concentrations in *Msm*.

Consistent with the induction of *recA*, the expression of the SOS gene *dnaE2* (the functional homologue of *E.coli dinB*) encoding the error-prone DNA polymerase was also significantly increased at the tested H_2_O_2_ concentrations [39]. However, unlike the expression pattern of *recA*, the expression level of *dnaE2* is independent of H_2_O_2_ concentrations, shown by the sustained magnitude of induction (40- to 75-fold, *P *< 0.0001) at the tested H_2_O_2_ concentrations. This is intriguing when considering that the expression of *dinB* was not markedly increased in *E.coli* upon exposure to 1 mM H_2_O_2_, while the SOS genes (*recA*, *recN*, *lexA*, and *dinD*) exhibited moderate induction (fold change < 10) [38]. The mechanism underlying the observed difference of transcriptional regulation of error-prone polymerase upon exposure to H_2_O_2_ remains unclear. Nevertheless, given that error-prone lesion bypass is critical for preventing lethal DNA damage, the remarkable induction of *dnaE2* may contribute to mycobacterial intrinsic resistance to mode-one killing [11,39].

Owing to chelation of Fe^2+^ by triphosphates, free nucleotides are also frequently oxidized by H_2_O_2_, which can lead to mutagenesis and DNA damage upon mis-incorporated into DNA [3,13,40] (Figure 3A). Our previous study demonstrated that sanitization of oxidized dCTP by MazG contributes to mycobacterial defence against oxidative stress [3,40,41]. The qRT-PCR results showed that *mazG* exhibited a “bell-curve” pattern of induction at the H_2_O_2_ concentrations varying from 1 to 5 mM (1.7- to 3.3-fold, *P *< 0.05). At 10 mM H_2_O_2_, expression of *mazG* was downregulated (Figure 3D), indicative of an intricate crosstalk among different transcriptional regulators [42]. Induction of *mazG* under oxidative stress may alleviate the mutagenesis and DNA damage caused by misincorporation of oxidized dNTPs (Figure 3A)[3].

### Metabolic adaptation

ROS-mediated metabolic deficiency mainly stems from the inactivation of [4Fe–4S] clusters of metabolic enzymes [8–10] (Figure 2A). Given that the generation of ROS is an inevitable process occurring in normal aerobic metabolism, it is not surprising that bacteria have evolved specialized metabolic enzymes and/or adaptation tactics to deal with oxidative stress. For example, in *E.coli* exposed to redox-cycling compounds, fumarase C, a non-Fe–S enzyme, and ROS-resistant aconitase A, were induced to replace the highly sensitive housekeeping counterparts to restore the TCA flux [43,44]. While in mycobacteria, accumulating evidence demonstrates that metabolic enzymes contribute to antioxidant defence and pathogenesis [2,5,18,22,45,46].

**Figure 4:**
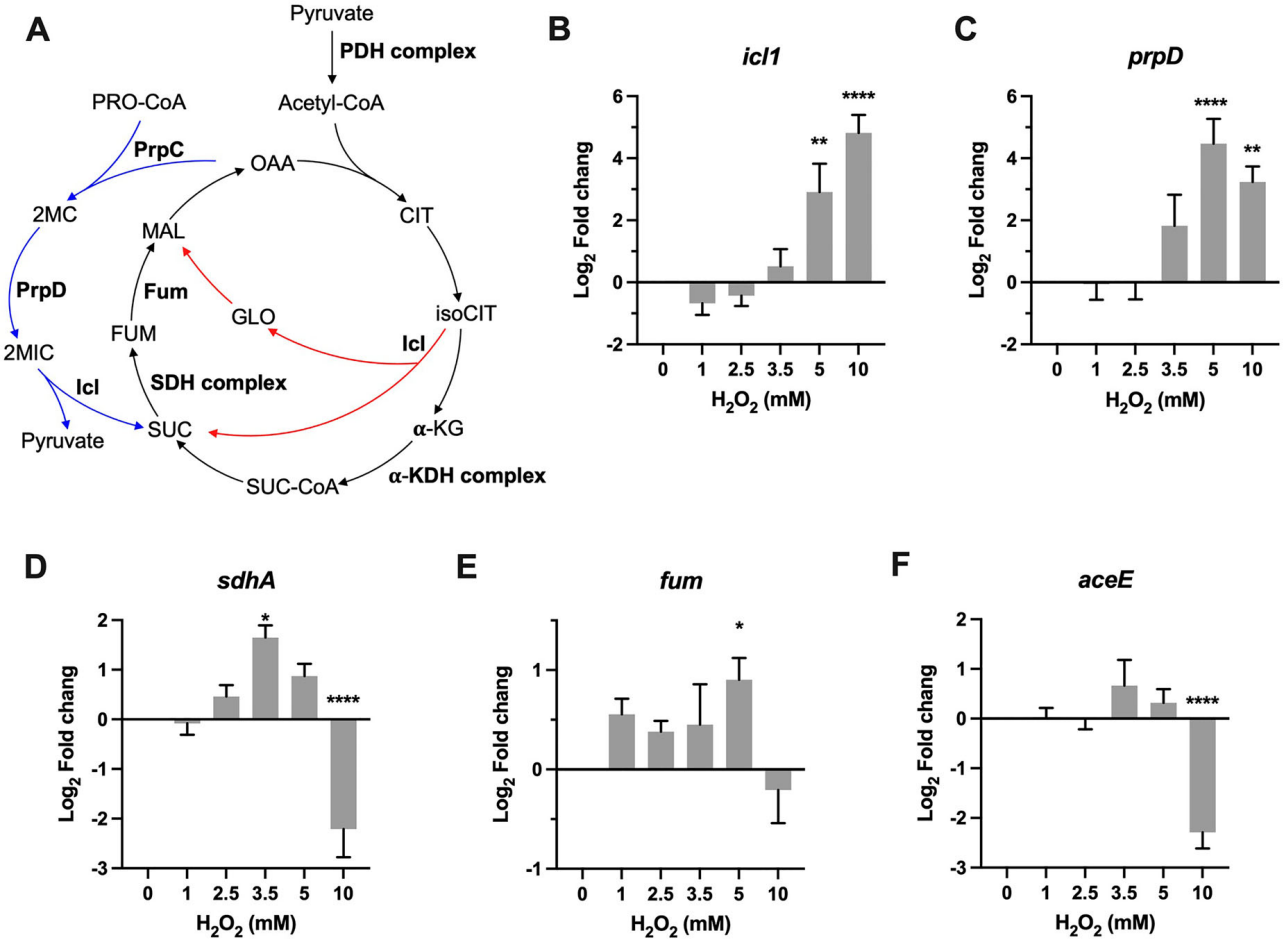
. Transcriptional profiles of genes encoding for metabolic enzymes involved in antioxidant defense. (A) Illustration of TCA, glyoxylate shunt, and methylcitrate cycle. Enzymes or enzyme complex involved in antioxidant response were shown as bold. PDH, pyruvate dehydrogenase; CIT, citrate; isoCIT, isocitrate; α-KG, α-ketoglutarate; α-KDH, α-ketoglutarate dehydrogenase; SUC-CoA, succinyl-CoA; SUC, succinic acid; SDH, succinate dehydrogenase; FUM, fumarate; MAL, malic acid; OAA, oxaloacetate; GLO, glyoxylate; PRO-CoA, propionyl-CoA; 2MC, 2 methylcitrate; 2MIC, 2 methylisocitrate. (B–F) Expression of *icl*, *prpD, sdhA, fum*, and *aceE* in *Msm* exposed to H_2_O_2_ for 50 min. Transcript levels were measured by the qRT-PCR, normalized relative to *sigA*, and expressed as Log_2_ fold change from untreated cultures. Data shown are mean ± SE with at least three independent experiments. *P < 0.05, **P < 0.01, ****P < 0.0001.

To probe the metabolic adaptation under different intensities of oxidative stress, we first examined transcriptional profiles of *icl1* and *prpD*, both are well-characterized antioxidant enzymes belonging to glyoxylate shunt and methylcitrate cycle (Figure 4A) [5,18]. The qRT-PCR data demonstrated that *icl1* and *prpD* exhibited a very similar expression pattern, shown by unchanged expression at 1 and 2 mM H_2_O_2_, followed by steadily increased induction by high H_2_O_2_ concentrations (Figure 4B-C). The expression of *icl1* was significantly induced 33-fold (*P *< 0.01) by 5 mM H_2_O_2_ and 43-fold (*P *< 0.0001) by 10 mM H_2_O_2_. The induction of *prpD* began at 3.5 mM H_2_O_2_ (6-fold) and approached maximal at 5 mM H_2_O_2_ (43-fold, *P *< 0.0001). These transcriptional profiles indicate that mycobacterial defence against high level of ROS implicates redirection of metabolism by ICL and PrpD.

ICL conducts the first committed step in the glyoxylate shunt, which implicates anaplerosis of TCA cycle intermediates and gluconeogenesis. In mycobacteria, ICL also functions as methylisocitrate lyase belonging to the methylcitrate cycle [47], a pathway involved in the metabolism of propionyl-CoA generated through β-oxidation of odd-chain fatty acids or degradation of branched-chain amino acids and cholesterol [18,48]. Because both glyoxylate shunt and methylcitrate cycle can generate succinate, we therefore measured the expression of *sdhA* and *fum,* which encode for succinate dehydrogenase A and a non-Fe–S fumarase, respectively (Figure 4A). As shown in Figure 4D, *sdhA* exhibited a “bell-curve” pattern of induction at the H_2_O_2_ concentrations varying from 1 to 5 mM, with the maximal induction at 3.5 mM H_2_O_2_ (3.2-fold, *P *< 0.05). Expression of *fum* was slightly increased (1.3- to 2.0-fold, *P *< 0.05) and sustained at the H_2_O_2_ concentrations varying from 1 to 5 mM (Figure 4E). In *Mtb*, Fum deficiency increases susceptibility to H_2_O_2_ [45]. Taken together, the induction of *sdhA* and *fum* by intermediate H_2_O_2_ concentrations may be indicative of maintenance of the TCA flux, a mechanism deployed by *E.coli* to defence against ROS [43,44].

The most intriguing aspect of these expression profiles was the reciprocal expression pattern between *icl1*/*prpD* and *sdhA*/*fum* at 10 mM H_2_O_2_ (Figure 4B–E). The maximal induction of *icl1*/*prpD* and sudden repression of *sdhA*/*fum* at 10 mM H_2_O_2_ perhaps reflect metabolic adaptation that may result in increased catabolism of propionyl-CoA or accumulation of succinate. In addition, the repression of *sdhA*, a FAD-dependent Fe–S-containing respiratory component, could also reduce the generation of endogenous ROS [11]. Previous study demonstrated that *Mtb* could slow and remodel its TCA cycle to increase production of succinate, which is used to flexibly sustain membrane potential, ATP synthesis, and anaplerosis, in response to varying degrees of O_2_ limitation [46]. However, the role of succinate in the antioxidant response remains elusive. To further probe whether *Msm* also slows its TCA cycle under intensified oxidative stress, we measured the expression of *aceE*, which encodes for a key component of pyruvate dehydrogenase complex (Figure 4A), and the results showed that the expression of *aceE* was significantly downregulated by 4.2-fold (*P *< 0.0001) at 10 mM H_2_O_2_ (Figure 4F). Given that glycerol is the major carbon source for *Msm* cultured *in vitro*, the repression of *aceE* may likely reflect a decreased fuelling of TCA cycle. Together, these results indicate that mycobacterial metabolic adaptation to ROS is strikingly dependent on the intensity of oxidative stress, and mycobacteria tend to shut down the TCA cycle and increase the activities of glyoxylate shunt and methylcitrate cycle to defence against intensified oxidative stress.

### Protein repair and degradation

We examined the transcription dynamics of genes linked to the well-characterized protein repair or degradation/recycling pathways to probe how they responded to oxidative stress. We have focused on the genes encoding for peptide methionine sulfoxide reductase (*msr*) and protease complex including proteasome and Clp system.

**Figure 5:**
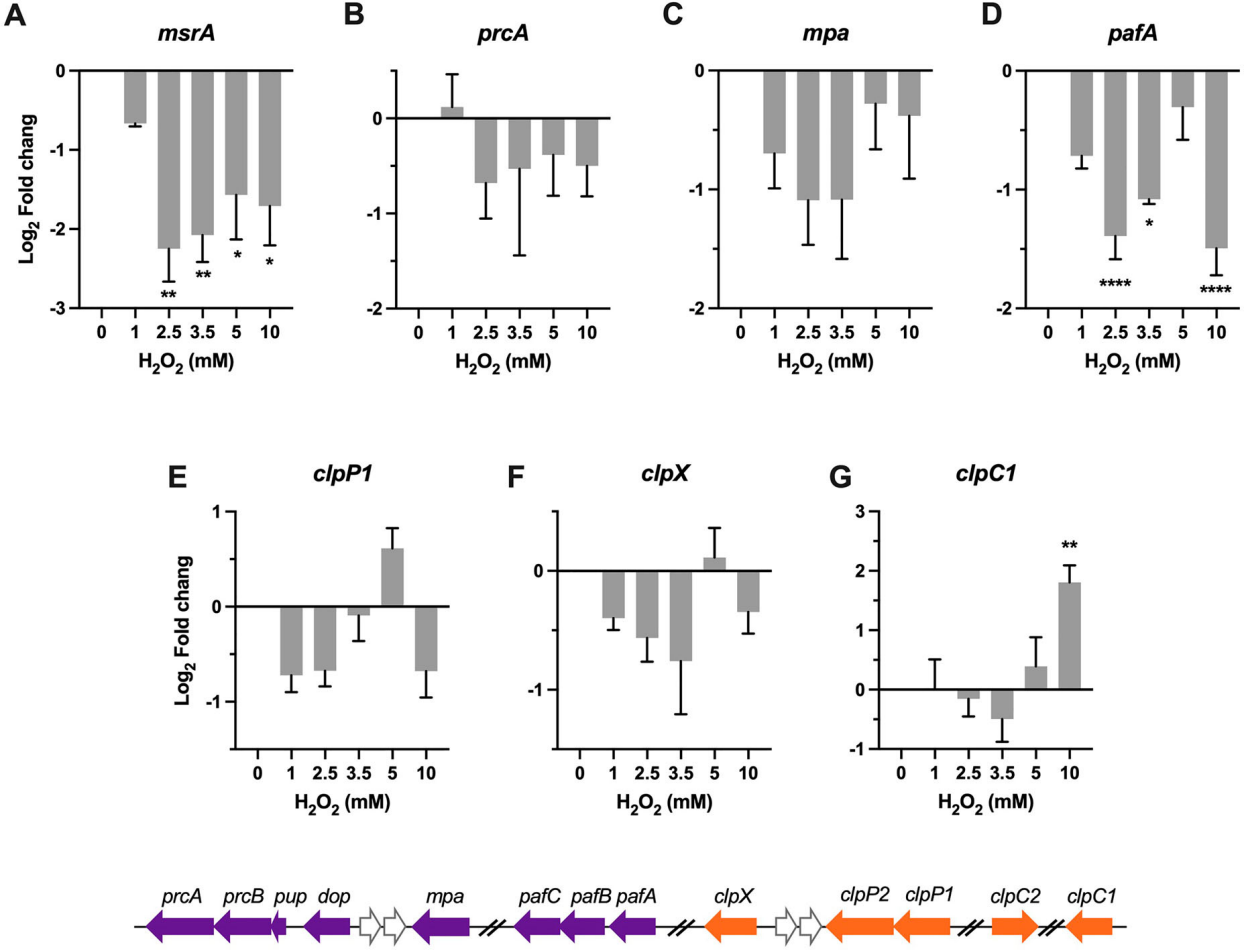
. Transcriptional profiles of genes encoding for protein repair and degradation. Expression of *msrA*, *prcA, mpa, pafA, clpP1, clpX, and clpC1* in *Msm* exposed to H_2_O_2_ for 50 min. Transcript levels were measured by the qRT-PCR, normalized relative to *sigA*, and expressed as Log_2_ fold change from untreated cultures. Data shown are mean ± SE with at least three independent experiments. *P < 0.05, **P < 0.01, ****P < 0.0001.

Oxidized methionine can be repaired by methionine sulfoxide reductase Msr. Previous studies demonstrated that deletion of *msrA* in either *Msm* or *E.coli* resulted in hypersensitivity to H_2_O_2_ [49,50]. However, our qRT-PCR results showed that the expression of *msrA* was significantly downregulated 1.6- to 4.3-fold (*P *< 0.05) upon exposure to the tested H_2_O_2_ concentrations (Figure 5A). Intriguingly, transcriptional repression was also observed on the genes encoding for the proteasome system (Figure 5B-D). Specifically, while expression of unfoldase *mpa* and *prcA*, which encodes for the α subunit of the proteolytic component and located in the *pup*-*prcB*-*prcA* operon, was slightly reduced (Figure 5B), the expression of the Pup (prokaryotic ubiquitin-like protein) ligase PafA were significantly and markedly downregulated (Figure 5C-D). Given that Mpa and PafA work together to recognize, unfold, and translocate the substrate proteins into the proteolytic chamber [51], these transcriptional profiles are indicative of active reduction of mycobacterial proteasome activity upon exposure to H_2_O_2_. According to the previous study showing that the *mpa*-, *pafA*-, and *prcBA*-deficient strains became more resistant than the wild-type *Mtb* to H_2_O_2_ [52], it appears that the observed repression of proteasome components may protect mycobacteria from H_2_O_2_. The mechanism underlying the protective effect of diminished proteasome activity remains undefined, however, it might associate with the regulatory role of proteasome on maintaining metal homeostasis (Cu^2+^, Fe^2+^, and Zn^2+^)[51].

The mycobacterial Clp system is the essential proteolytic system composed of the proteolytic subunits, ClpP1 and ClpP2, and the ATPase adapters, ClpX or ClpC1 [53,54]. A recent study established that the induction of *clpSA* (ClpA is orthologous to ClpC1) protected *E.coli* from endogenous H_2_O_2_ [19]. As shown in Figure 5E–G, while the expression of *clpP1* and *clpX* was slightly reduced or unchanged at most of tested H_2_O_2_ concentrations, the expression of *clpC1* was significantly increased at 10 mM H_2_O_2_ (4.2-fold, *P *< 0.01) (Figure 5G). Because the ATPase adapter determines substrate specificity of the Clp proteolytic process, the exclusive induction of *clpC1* may indicate a selective protein degradation process at high H_2_O_2_ concentrations. Previous study demonstrated that silencing of *clpC1* in *Mtb* resulted in accumulation of proteins mainly involved in intermediary metabolism, respiration, and lipid metabolism [55], suggests that the induction of *clpC1* under oxidative stress may associate with metabolic adaptation.

### Profiles of coordinated gene regulation

**Figure 6.**
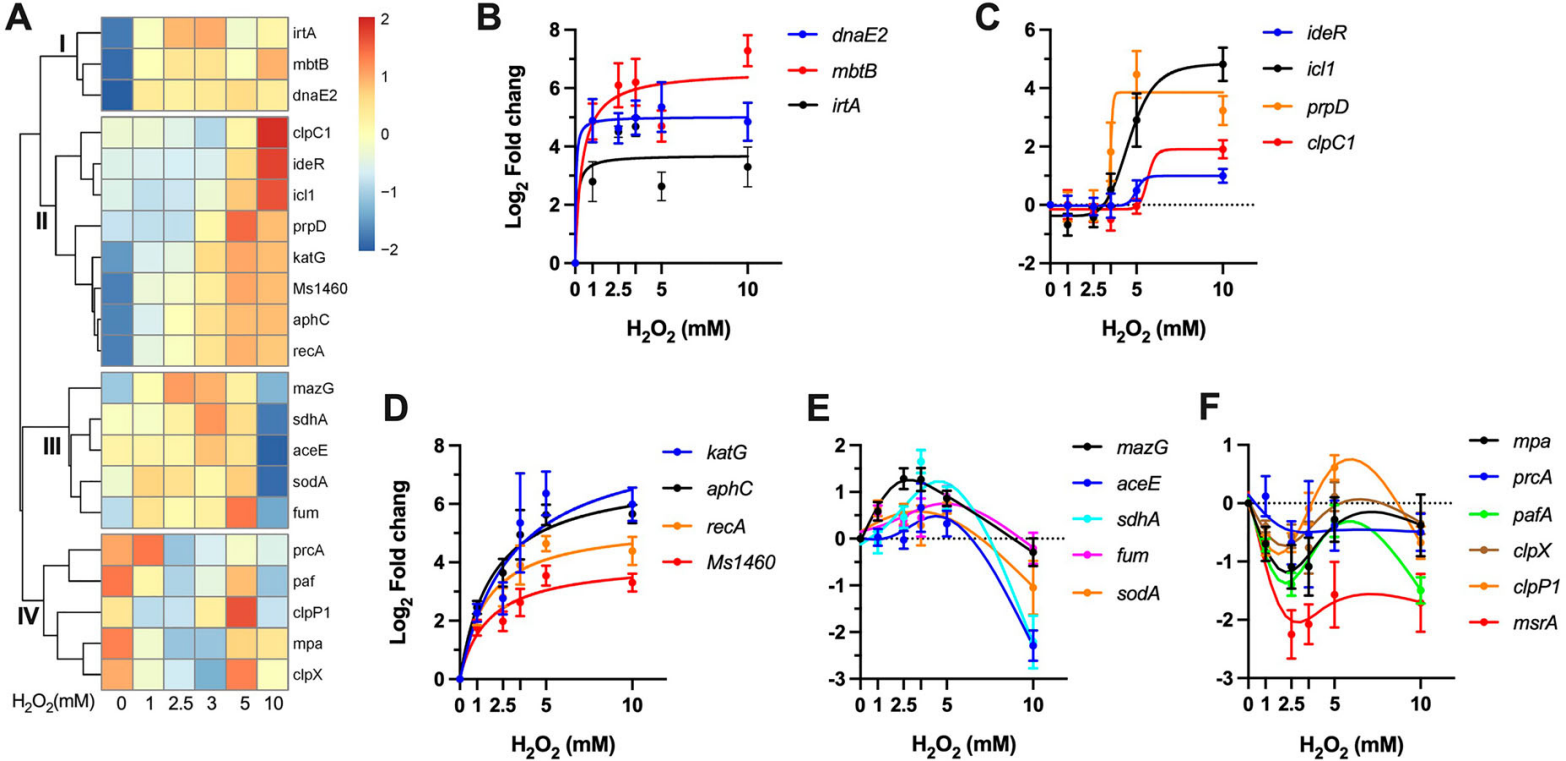
Profiles of coordinated gene regulation. (A) Heatmap showing the unsupervised hierarchical clustering based on the Log_2_ fold change values of tested genes. Colour shown represents Z-scored expression values. (B-F) H_2_O_2_ concentration-dependent expressional patterns of clustered genes. Data shown are mean ± SE with at least three independent experiments.

To interrogate the physiological states and survival mechanisms under different intensities of oxidative stress, we performed cluster analysis of the H_2_O_2_ concentration-dependent dynamics of gene expression. As shown in Figure 6A, four clusters of coordinated gene expression were identified.

The cluster I includes three genes showing sustained level of induction throughout the tested H_2_O_2_ concentrations (Figure 6B). This subset includes genes involved in error-prone DNA synthesis (*dnaE2*) and iron acquisition (*mbtB*, *irtA*). The sustained expression of these genes at different intensities of oxidative stress is well consistent with the notion that DNA and iron are the primary targets of ROS [11,16]. Moreover, this expression profile also indicates that mycobacteria may deploy increased iron acquisition and error-prone lesion bypass as fundamental strategies to combat oxidative stress, which is distinct from the defence tactics of *E. coli*, characterized by shrinking the iron pool and delaying the DNA repair [11,16].

The cluster II shows an expression pattern of H_2_O_2_ concentration-dependent increasing (Figure 6A). Based on the dose-dependence, this subset genes can be further grouped into two classes. The first class includes *ideR*, *icl1*, *prpD*, and *clpC1*(Figure 6C), which exhibited unchanged expression at low H_2_O_2_ concentrations (1–3.5 mM) and sudden induction at high concentrations (5 or/and 10 mM), indicative of responses specific to stimuli induced by intensified oxidative stress. The most intriguing feature of this class is the enrichment of genes involved in metabolic pathways, including methylcitrate cycle (*icl1*, *prpD*) and glyoxylate shunt (*icl1*). In addition, recent study also established that *clpC1* mainly targets proteins involved in metabolism [55]. Thereafter, it appears that metabolic remodeling may play an important role in mycobacterial defence against high level of ROS. The second class is characterized by steadily increased expression of *katG*, *aphC, recA*, and the Suf system (Figure 6D), demonstrating that the expressions of the H_2_O_2_ scavenging enzymes, DNA damage response, and Fe–S cluster repair function were well correlated to the intensity of oxidative stress.

The cluster III contains a geneset (*mazG*, *aceE*, *sdhA*, *fum*, and *sodA*) that exhibited a “bell-curve” pattern of expression (Figure 6E), suggesting an intricate crosstalk among transcriptional regulators under different intensities of oxidative stress. This profile was characterized by steadily increased induction by H_2_O_2_ below 5 mM and sudden repression by 10 mM, suggesting that the bacilli may encounter novel pressures that requires additional bacterial adaptation. This class also showed enrichment of genes involved in metabolism, including TCA cycle (*sdhA*, *fum*), pyruvate metabolism (*aceE*), and nucleotide metabolism (*mazG*). These data indicate that mycobacteria can sophistically remodel its metabolism to adopt to different intensities of oxidative stress (Figure 6C,E).

The cluster IV is exclusively composed of genes involved in protein degradation and repair (Figure 6F), which exhibited repressed (*msrA* and *pafA*) or unchanged expression. The repression of *pafA* might provide an antioxidant effect, given that the *mpa*-, *pafA*-, and *prcBA*-deficient strains were shown to be more resistant than wild-type *Mtb* to H_2_O_2_ [52]. The role of mycobacterial Clp system in antioxidant response remains unclear. However, the remarkable upregulation of *clpC1* and baseline expression level of *clpP1* and *clpX* at 10 mM H_2_O_2_ imply that mycobacterial Clp system might participate in antioxidant defence at intensified oxidative stress by selective protein degradation [19].

## Conclusions

Given that bacterial transcriptional profiles could mirror the physiological states and survival mechanisms, this study offers several unique insights into mycobacterial antioxidant defence strategies under different intensities of oxidative stress. The sustained induction of *mbtB* and *irtA* by H_2_O_2_ indicates that *Msm* upregulates the iron acquisition upon oxidative stress. Importantly, these transcriptional signatures were in line with the observations in *Mtb* upon exposure to H_2_O_2_ or during infection of mice, shown by upregulation of *mbtB* and downregulation of *bfrA* (encoding an iron-storing bacterioferritin) [20,56]. This is strikingly different from the defence strategy of *E. coli* characterized by shrinking the iron pool and delaying the DNA repair [16]. Given that iron plays a critical role in oxidative damages to DNA, the difference on iron metabolism between mycobacteria and *E.coli* may substantially affect DNA damage and the SOS response as observed in this study. Although mycobacteria were phenotypically refractory to DNA damage-dependent mode-one killing caused by low concentration of H_2_O_2_ (1–2 mM) [20,37], our results indicated that DNA damage occurs under these conditions, as shown by the marked induction of *recA* by 1–2 mM H_2_O_2_. Therefore, it is tempting to speculate that mycobacteria could prevent the killing events downstream of DNA damage by low concentrations of H_2_O_2_. Among the various types of DNA damage, DSBs are the major reason accounting for massive cell death. In *Mycobacterium*, DSBs could be directly repaired through homologous recombination, nonhomologous end-joining, and single-strand annealing (SSA) [57]. Mycobacteria could also deploy translesion DNA polymerase (DnaE2) to prevent DSBs by allowing bypass of lethal replication-blocking lesions [39]. Based on this, it appears that the remarkable and sustained induction of *dnaE2* (40- to 75-fold) and *recA* by H_2_O_2_ might contribute to mycobacterial survival of mode-one killing. In this regard, it is worth noting that *dinB* (functional homologue of *dnaE2*) was not markedly induced in *E.coli* exposed to 1 mM H_2_O [38]. Together, these results suggest that mycobacteria have evolved an adaptative strategy that could simultaneously upregulate the iron acquisition and DNA repair systems to meet the need for repair of Fe-cofactored enzymes and DNA maintenance (refractory to mode-one killing) under oxidative stress.

The dynamic alternations of metabolic gene expression, *e.g.* the “bell-curve” expression pattern of metabolic genes (*aceE*, *sdhA*, *fum*) and the reciprocal expression of genes belong to different metabolic pathways (TCA *vs* methylcitrate cycle and glyoxylate shunt), indicate that mycobacterial metabolic response to ROS is strikingly dependent on the intensity of oxidative stress. Given that the Fe–S enzymes (aconitase, SdhA, FumA) of TCA cycle are highly sensitive to ROS, the upregulation of *sdhA* and *fum* (encodes a non-Fe–S fumarase C) by intermediate H_2_O_2_ concentrations (3.5–5 mM) may reflect a compensatory response to restore the metabolic activities. Induction of *fumC* and ROS-resistant aconitase A was also observed in *E.coli* upon exposed to ROS [43,44], suggesting that both species deploy similar strategy to maintain the TCA flux. The reciprocal expression of genes between TCA and methylcitrate cycle/glyoxylate shunt at 10 mM H_2_O_2_ suggests that redirection or remodel of metabolic pathways underlies mycobacterial adaptation to intensified oxidative stress. For instance, the remarkable induction of *icl1*/*prpD* and the sudden repression of *aceE*/*sdhA*/*fum* by 10 mM H_2_O_2_ may reflect the redirection of carbon flux from TCA cycle to methylcitrate cycle and glyoxylate shunt. Induction of *icl1* and/or *prpD* was also observed in *Mtb* upon exposure to H_2_O_2_ or bactericidal antibiotics, as well as during infection of mice [5,20,56], indicating that these conditions could induce similar physiological transition and adaptation in mycobacteria.

## Materials and methods

### Bacterial strains and growth conditions

*Mycolicibacterium smegmatis* strain mc^2^-155 was grown at 37°C, 110 rpm in 7H9 broth (Difco) containing 0.2% (*v/v*) glycerol and 0.05% (*v/v*) Tween 80. Three colonies were mixed and inoculated into 2 ml 7H9 and grown at 37°C to reach a cell density of OD_600_∼2. The seeding culture was diluted 1:100 in 100 ml 7H9 broth (in a 500-ml flask) and grown to exponential phase (OD_600 _= 0.5–0.6) at 37°C with shaking at 110 rpm. Exponential-phase cultures were split into 10-ml aliquots (in a 15-ml centrifuge tube); one aliquot was left untreated, and the other aliquots were treated with the indicated concentrations of hydrogen peroxide statically at 37°C for 50 min [30,41,58]. The H_2_O_2_ concentrations indicated in this study were validated by the Peroxide Assay kit (Sigmaaldrich).

### RNA isolation

Culture aliquots were harvested by centrifugation at 3200×*g* for 5 min at room temperature. The supernatant was discarded, and the cell pellet was immediately resuspended in 1 ml cold TRIzol (Invitrogen), transferred to 2-ml screw cap tubes containing 500 μl of 0.1 mm zirconia-silicate beads. Cells were mechanically disrupted by bead beating (Minilys, Bertin) for five cycles (35 s at maximal speed) with cooling on ice for 1 min between pulses. The cell lysates were centrifuged at 16,000 × *g* for 5 min at 4 °C, and the supernatant was transferred to a new centrifuge tube containing Phase Lock Gel (TIANGEN). Three hundred microliter of chloroform was added to the supernatant and mixed vigorously for 30 s. The mixture was incubated at room temperature for 10 min, followed by centrifugation at 20,000 × g for 30 min at 4°C. The supernatant was transferred to a new microcentrifuge tube, mixed with an equal volume of isopropanol immediately by gently inverting 10 times, placed on ice for 30 min, followed by centrifugation at 20,000 × g for 30 min at 4°C. The supernatant was discarded, and the pellet was washed by 1 ml of 70% ethanol, followed by centrifugation at 20,000 × g for 10 min at 4°C. The pellet was dried at room temperature. RNA samples were treated with 10 U RNase-free DNaseI (NEB) for 30 min at 37°C, further purified using the GeneJET RNA purification kit (Thermofisher). RNA yields were quantified by Nanodrop (Thermo Scientific, Waltham, MA, USA). RNA quality was assessed by the agarose gel electrophoresis. RNA was subjected to the PCR to confirm lack of residual genomic DNA.

### qRT-PCR

cDNA was synthesized using the SuperScript III First Strand kit (Invitrogen) with random hexamer primer, according to the manufacturer’s instructions. qRT-PCR was carried out using TB Green^®^ Premix Ex Taq GC (TaKaRa). Gene expression data were normalized to *sigA*. Relative gene expression was calculated using the 2^−ΔΔCt^ method. The primers used for the qRT-PCR were described in Table S2.

### Statistical analyses

Significance tests were performed in the GraphPad Prism 9 (GraphPad Software, San Diego, CA, USA) using a one-way analysis of variance (ANOVA) and a Dunnett posttest. A statistical difference between the control (untreated) and another is marked above the column (*P < 0.05; **P < 0.01; ***P < 0.001; ****P < 0.0001).

## Supplementary Material

Supplemental Material

Supplemental Material
