# Supplementary material for: H2O2 concentration-dependent kinetics of gene expression: linking the intensity of oxidative stress and mycobacterial physiological adaptation
Source: Emerg Microbes Infect. 2022 Feb 16;11(1):573–84. doi: 10.1080/22221751.2022.2034484 (PMC8856045; doi:10.1080/22221751.2022.2034484)
Supplement: Supplemental Material [file TEMI_A_2034484_SM8764.docx]

**Table S2. Primers used in this study**

| **Primers** | **Sequences** |
| --- | --- |
| sigA F | GACGACGACATCGACGAG |
| sigA R | CAGCTCCACCTCTTCTTCG |
| sodA F | AGCTGCACCACAGCAAGCA |
| sodA R | ATCGAGTGGTTGATGTGGCCA |
| katG F | AAGTACGGCAAGAACCT |
| katG R | TTGTCGTCGAGCCATT |
| aphC F | GGTGACCAGTTTCCGGAGTA |
| aphC R | TCGAAGTCCTCGTTCAGCTT |
| mbtB F | AGTTGCAGGGTGAGGTCTTC |
| mbtB R | AGGTGCCCGAGTTGTACAG |
| irtA F | GAGAACGACCGGCTCATT |
| irtA R | AAACCGAATTCGTCGCGCA |
| ideR F | AGCGCGACGGTCTGTTGCA |
| ideR R | CTCGCTCATGACGTGCTCC |
| Ms1460 F | TTCCTGCTGCCGACGAG |
| Ms1460 R | CGCCTCGATCAATGCGTC |
| recA F | GAGATCGAGGGCGAGATG |
| recA R | GGCGTAGAACTTCAGTGCCT |
| dnaE2 F | CAGGTTCTACGACGGGATG |
| dnaE2 R | CGAAGCTCAGCGAATGACT |
| mazG F | ACACCATCCGTTCAGCATC |
| mazG R | GCCTTGACCTTCTTCTCCTG |
| aceE F | TGAGTGGCTCGAATCCTTCG |
| aceE R | AACCACGGTTCCAGCTC |
| sdhA F | TGCAGCAGTCCATGGA |
| sdhA R | AGGAAGCCAAGCTCGAT |
| fum F | GCTGGAGGTCCTGCACGCAT |
| fum R | ACGTGGCAGCGTGGCCTTGA |
| prpD F | GCTACGGCGTACGAGATCCAGA |
| prpD R | TCGTCAGGTGCAGAGCCTG |
| icl1 F | GACCAGAGCCTGTAC |
| icl1 R | GCAGCTCGTAGACGTTG |
| clpP1 F | ATGGCGATCTACGACACCAT |
| clpP1 R | ATCAGGATGCGCGCAT |
| pafA F | GCGGAATGCGACAACCTGATA |
| pafA R | TGGCAGCCGTAGGAGTT |
| msrA F | ATCCGACGTACCGCAAT |
| msrA R | ACGTCGTTGCCCTGA |
| clpX F | AAGAGCGAGAACCCGTCGATC |
| clpX R | AACACGTTGGTGGTGTCGATC |
| mpa F | TCACCCAACATCGAGGTCAA |
| mpa R | CGAAGCGTGCTGATCTCAC |
| prcA F | ACGTGACGGGCCGACAGCT |
| prcA R | AAGTGCGGCTCGTCGGCGAT |
| clpC1 F | ACTTCAAGAACACGGTGCTG |
| clpC1 R | CTCGTAGTTGTTCTCGCTGC |
