# Supplementary material for: H2O2 concentration-dependent kinetics of gene expression: linking the intensity of oxidative stress and mycobacterial physiological adaptation
Source: Emerg Microbes Infect. 2022 Feb 16;11(1):573–84. doi: 10.1080/22221751.2022.2034484 (PMC8856045; doi:10.1080/22221751.2022.2034484)
Supplement: Supplemental Material [file TEMI_A_2034484_SM8758.docx]

**Table S1. Genes selected in this study**

| **Genes** | **Function** | **Locus tag**  **in Msm mc^2^ 155** | **Locus tag**  **in Mtb H37Rv** | **Essentiality** |
| --- | --- | --- | --- | --- |
| *sigA* | primary housekeeping sigma-factor of mycobacteria | MSMEG_2758 | Rv2703 | Essential |
| *sodA* | Superoxide dismutase [Fe] SodA | MSMEG_6427 | Rv3846 | Essential |
| *katG* | Catalase-peroxidase-peroxynitritase T | MSMEG_6384 | Rv1908c | Non-essential |
| *aphC* | Alkyl hydroperoxide reductase C | MSMEG_4891 | Rv2428 | Non-essential |
| *mtbB* | Phenyloxazoline synthase, involved in the biogenesis of siderophore mycobactins | MSMEG_4515 | Rv2383c | Non-essential |
| *irtA* | Iron-regulated transporter IrtA | MSMEG_6554 | Rv1348 | Essential |
| *ideR* | Iron-dependent repressor of siderophore biosynthesis and iron uptake | MSMEG_2750 | Rv2711 | Essential |
| *Rv1460* | transcriptional regulatory protein, co-transcribed with Rv1461-Rv1461 | MSMEG_3121 | Rv1460 | Non-essential |
| *recA* | Involved in regulation of nucleotide excision repair, in genetic recombination, and in induction of the sos response | MSMEG_2723 | Rv2737c | Non-essential |
| *dnaE2* | Error-prone DNA polymerase, functional ortholog of E.coli dinB | MSMEG_1633 | Rv3370c | Non-essential |
| *mazG* | NTP pyrophosphohydrolase | MSMEG_5422 | Rv1021 | Non-essential |
| *aceE* | Pyruvate dehydrogenase E1 component | MSMEG_4323 | Rv2241 | Non-essential |
| *sdhA* | succinate dehydrogenase | MSMEG_1670 | Rv3318 | Non-essential |
| *fum* | class II fumarate hydratase | MSMEG_5240 | Rv1098c | Essential |
| *prpD* | 2-methylcitrate dehydratase | MSMEG_6645 | Rv1130 | Non-essential |
| *icl1* | isocitrate lyase | MSMEG_0911 | Rv0467 | Essential |
| *clpP1* | ATP-dependent CLP protease proteolytic subunit 1 | MSMEG_4673 | Rv2461c | Essential |
| *pafA* | Proteasome accessory factor A | MSMEG_3890 | Rv2097c | Non-essential |
| *msrA* | peptide methionine sulfoxide reductase | MSMEG_6477 | Rv0137c | Non-essential |
| *clpX* | ATP-dependent Clp protease | MSMEG_4671 | Rv2457c | Essential |
| *mpa* | Mycobacterial proteasome ATPase | MSMEG_3902 | Rv2115c | Non-essential |
| *prcA* | Proteasome alpha subunit PrcA, assembles with beta subunit PrcB | MSMEG_3894 | Rv2109c | Essential |
| *clpC1* | ATP-dependent protease ATP-binding subunit | MSMEG_6091 | Rv3596c | Essential |
